# The Relationship between Self-Rated Health and Physical Fitness in Polish Youth

**DOI:** 10.3390/healthcare12010024

**Published:** 2023-12-21

**Authors:** Maciej Kochman, Aleksandra Kielar, Marta Kasprzak, Krystian Maruszczak, Wojciech Kasperek

**Affiliations:** Physiotherapy Department, Institute of Health Sciences, College of Medical Sciences, University of Rzeszów, 35-215 Rzeszów, Poland

**Keywords:** well-being, physical activity, cardiorespiratory fitness, adolescents, children, sleep, postural disorders, self-perceived health

## Abstract

Self-rated health (SRH) is a tool for assessing a population’s health across the lifetime, and seems to be a dynamic assessment of current health status and a strong predictor of cardiovascular disease and mortality, whereas insufficient levels of physical fitness in adolescence are a significant health problem and may contribute to the development of many disorders in adulthood. In this cross-sectional study, we attempted to assess the relationship between SRH and the physical fitness of adolescents. Two hundred and thirty-five adolescents (eighty-five boys and one hundred and fifty girls) aged 16–17 were recruited for this study. The study procedures included a short author questionnaire and physical fitness assessment (Zuchora’s Physical Fitness Index). Boys declared better health states and reported less frequent morbidity of seasonal diseases (*p* < 0.05). No differences were found in physical fitness, the incidence of postural disorders, lower limb malalignments, foot deformities, sleep duration, and perception of physical activity on physical condition and well-being in boys and girls (*p* > 0.05). The comparison of physical fitness levels in adolescents with different SRH, sleep duration, perceptions of physical activity on physical condition and well-being also showed significant differences (*p* < 0.05). Positive SRH and perception of physical activity on physical condition and well-being, proper sleep duration (7–8 h/night), and a lack of foot deformities are associated with a better physical fitness in adolescents. Physical fitness seems to be a good predictor of SRH only in Polish boys, but not girls and the entire population. The lack of significance in the entire population could be attributed to the substantial number of girls in the study group. Despite that, it is recommended to encourage adolescents to engage in regular exercises, sleep routines and healthy lifestyles. Further research should be based on a more representative group, with a comparable number of girls and boys in the study group and potential confounders, but also they should be focused on SRH predictors to improve SRH in Polish girls.

## 1. Introduction

Self-rated health (SRH) is a subjective measure that involves a person’s feelings about their health status, including a few spheres, such as biological, psychological, functional, and social aspects, as well as health behaviors or beliefs. SRH is a crucial proxy for assessing a population’s health across the lifetime [1]. Despite the non-specific nature of SRH, it seems to be a dynamic assessment of current health status and a strong predictor of cardiovascular disease and mortality [2].

Numerous scientific reports show that regular physical activity leads to a broad spectrum of health benefits improving bone mineralization [3], reducing excessive fat accumulation [4], improving the efficiency of the respiratory system [5], and increasing strength and muscular endurance, as well as preventing obesity and cardiovascular problems [6,7]. Furthermore, regular physical activity improves the quality of sleep, mental health, and cognitive functioning [8,9]. On the other hand, lack of physical activity or its insufficient level is attributable to plenty of health problems. An inactive lifestyle contributes to type 2 diabetes, a higher prevalence of depression, lower self-esteem, increased prevalence of insulin resistance and the risk of metabolic and cardiovascular diseases [10]. There is a clear relationship between physical activity and physical fitness as physical fitness is a consequence of physical activity [11]. This is extremely important because physical fitness is related to health status and prevention of health complications [12,13].

Physical fitness is a set of individual features that are either health- or skill-related, and it refers to the ability to perform activities of daily living with vigor and alertness, without excessive fatigue, and with sufficient energy to enjoy leisure-time activities [14,15]. Many scientific reports show that a higher level of physical fitness produces health benefits in adolescence. There is a strong scientific evidence that adolescents with higher fitness levels have better memory performance, more efficient cognitive functions, stronger muscles, and a more efficient cardiopulmonary system compared to adolescents with lower fitness [12,13,14,15,16]. Furthermore, exercise intervention may reduce the risk of adverse cardiovascular incidents. This is because physical fitness plays a crucial role in promoting healthy vascular structure and function in adolescents [17].

The body of adolescents is susceptible to many factors determining health status and, among them, physical fitness is one of the most influential ones [18]. It is also well known that the changes related to the transition from adolescence to adulthood may have important implications for health in adulthood. The scientific literature confirms that developing healthy habits in that period affects health during adulthood [19]. The motivation to undertake this research was the fact that insufficient levels of physical fitness in adolescence are nowadays a significant health problem, and this may contribute to the development of many disorders in adulthood. Furthermore, we did not find any Polish study that analyzed the relationship between SRH and physical fitness, especially in adolescents. There are studies concerning the association between SRH and physical activity or lifestyle; those were, however, focused on older populations [20,21]. Therefore, the objective of this cross-sectional study was to assess the relationship between SRH and physical fitness of Polish adolescents. Based on that, we wanted to answer the following research questions: (1) Are there differences in the physical fitness between boys and girls? (2) Are there relationships between gender and SRH, the incidence of seasonal diseases, postural disorders, lower limb malalignments, foot deformities, sleep duration, and perception of physical activity on physical condition and well-being? (3) Does physical fitness depend on SRH, sleep duration, the perception of physical activity on physical condition and well-being, the incidence of postural disorders, lower limb malalignments, and foot deformities?

## 2. Materials and Methods

### 2.1. Study Design and Ethics Approval

This cross-sectional study was approved by the Bioethics Committee of the Medical University of Lublin (No. KE-0254/13/2018). The study was conducted in accordance with the Strengthening the Reporting of Observational Studies in Epidemiology (STROBE) guidelines.

### 2.2. Participants

**Figure 1:**
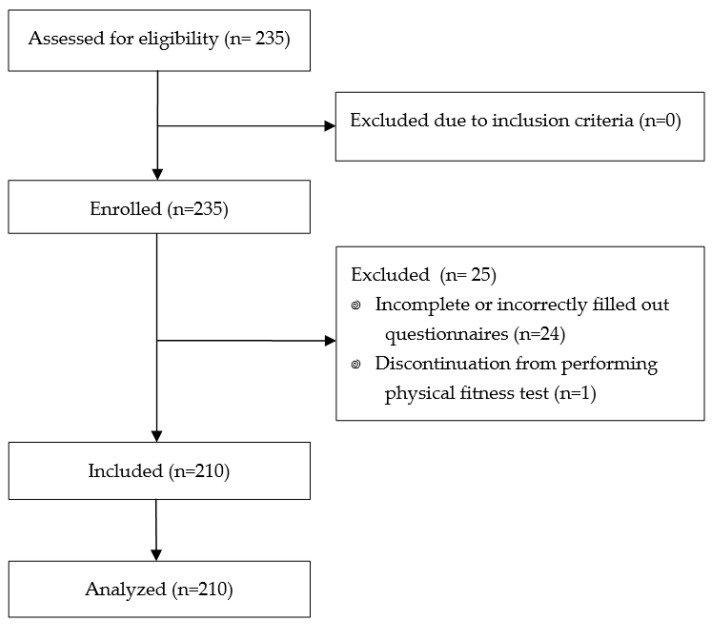
Participants flowchart.

For this study, two hundred and thirty-five adolescents, eighty-five boys (36.2%) and one hundred fifty girls (63.8%) aged 16–17 from five high schools in Rzeszów, Poland, were recruited. The inclusion criteria were as follows: (1) written informed consent to participate in the study; (2) age 16–17 years; and (3) present no contraindication to physical activity. The exclusion criteria were the following: (1) lack of informed consent; (2) chronic diseases, pain, injury, or other conditions within 6 months from the study indicating those as contraindications to perform physical exercises; (3) discontinuation from performing tests; and (4) incorrectly performed physical trials. Twenty-four participants returned incomplete or incorrectly filled out questionnaires, and one participant discontinued form performing physical fitness test; therefore, due to failure to complete all research procedures and missing data, twenty five participants were excluded. Finally, for further analysis, two hundred and ten participants were included (Figure 1).

Boys were characterized by higher mean values of body weight, height and BMI compared to girls. Study group characteristics are shown in Table 1.

### 2.3. Study Procedures

All study procedures were performed from September 2018 to May 2019 in the morning at the high school gyms by a qualified physiotherapist. Before the start of the investigation, all participants were informed about the aim of the study and had the opportunity to ask questions about it. The participants were also informed they can discontinue the study at any time and without reason. After that, all participants gave their written consent to participate in the study. The study was conducted in accordance with the Declaration of Helsinki. The study procedures included a short author questionnaire and physical fitness assessment using Zuchora’s Physical Fitness Index.

### 2.4. Research Questionnaire

Firstly, all participants filled out the short questionnaire regarding basic personal information, body weight and height, SRH, incidence of seasonal diseases, perception of physical activity on physical condition and well-being, and sleep duration, as well as medically confirmed diagnosis of postural defects, lower limb malalignments or foot deformities [22]. Participants assessed their SRH by answering one question: “How would you rate your health in general”, with the following possible answers: 1. very good, 2. good, 3. poor, and 4. bad. Due to an insufficient number of “bad” answers (n = 1), further analysis was conducted using three variables: “very good”, “good” and combined “poor or bad”. The incidence of seasonal diseases was evaluated by one question: “How often do you suffer from seasonal diseases (e.g., cold)”, with the following possible answers: 1. very often, 2. often, 3. rarely, 4. almost never. The perception of physical activity on physical condition was assessed by one question: “In your opinion, how your physical activity impacts your physical condition”, with the following possible answers: 1. definitely positively, 2. Irrelevant, 3. definitely negatively. The perception of physical activity on well-being was evaluated using one question: “In your opinion, how your physical activity impacts your well-being”, with the following possible answers: 1. definitely positively 2. irrelevant, 3. definitely negatively. Due to an insufficient number of “definitely negative” answers (n = 2), further analysis was conducted using 2 variables: “definitely positively” and combined “irrelevant or definitely negatively”. The sleep duration was assessed by one question: “How long do you sleep for on average on weekdays and weekend days?”, with the following possible answers: 1. less than 5 h, 2. 5–6 h, 3. 7–8 h, 4. 9–10 h, 5. more than 10 h. Due to an insufficient number of answers “less than 5 h” (n = 7) and “more than 10 h” (n = 2), further analysis was conducted using 3 variables: “6 h or less”, “7–8 h” and “9 h or more”. The occurrence of postural defects, lower limb malalignments, and foot deformities was evaluated by three questions: “Do you have a medically confirmed diagnosis of postural defects?”, “Do you have a medically confirmed diagnosis of lower limb malalignments?”, and “Do you have a medically confirmed diagnosis of foot deformities”, with “yes” or “no” possible answers.

### 2.5. Physical Fitness Assessment

Then, all participants were tested using Zuchora’s Physical Fitness Index. This index consists of trials evaluating arm and abdominal muscle strength, flexibility, endurance, speed and jumping ability. Each trial is evaluated using a 6-grade scale, and the maximum possible score for the test is 36. The higher the score of the test, the fitter and more physically advanced the participant is. The total score of this index can be analyzed by referring to the norms developed for age-specific groups, separately for males and females. A detailed description and evaluation of this index have been described in previous studies [23,24]. In this study, the endurance trial was abandoned.

### 2.6. Statistical Analysis

Statistical analysis was performed in Statistica 13.3. The Shapiro–Wilk test was performed to assess the normality distribution of data. The associations between categorical variables were assessed using Pearson’s Chi square test or the likelihood ratio Chi-square test. The differences between quantitative and normally distributed data were calculated using a t test. For not normally distributed data, the differences were calculated using the Mann–Whitney U test (for two groups) or the Kruskal–Wallis test (for three or more groups). Statistical significance was assumed if *p* < 0.05.

## 3. Results

The comparison of physical fitness levels showed no significant differences between boys and girls. This comparison is shown in Table 2.

The analysis of SRH between boys and girls revealed statistically significant relationships. Most girls declared “good” health, while most boys declared both “good” and “very good” health. In both groups, the least adolescents declared “poor or bad health”. Also, more boys than girls declared “very good” health, while more girls declared “good” or “poor or bad” health compared to boys.

The assessment of the incidence of seasonal diseases in boys and girls also indicated significant relationships between boys and girls. In both groups, most of the respondents declared that they “rarely” suffer from seasonal diseases. The least boys and girls declared “very often” incidences of seasonal diseases. More girls declared incidences of seasonal diseases “very often”, “often” and “rarely” compared to boys, while more boys declared “almost never” incidence of seasonal diseases.

We have found no significant associations between the incidence of clinically diagnosed postural disorders, lower limb malalignments, foot deformities, sleep duration, and perception of physical activity on physical condition and well-being in boys and girls. The above results have been shown in Table 3.

The comparison of physical fitness levels in boys and girls with different SRH showed significant differences only in boys. The mean value of physical fitness of boys who rated their health “very good” was significantly higher than those who rated their health “good” and “poor or bad”. We did not observe significant differences in girls and the entire population.

The analysis of physical fitness levels in boys and girls with different sleep duration revealed significant differences only in girls and the entire population. Both girls and the entire population who sleep 7 to 8 h received higher scores on physical fitness test, compared to those who sleep less than 6 h and more than 9 h. We did not observe significant differences in physical fitness in boys with different sleep durations.

The comparison of physical fitness in boys and girls with different perceptions of physical activity on physical condition showed significant differences only in boys and the entire population. Boys who reported a “definitely positive” influence of physical activity on physical condition received a higher score of physical fitness than those who reported “irrelevant” or “definitely negative” influence of physical activity on physical condition. Similar findings were observed when analyzing the results of the entire population. We have observed no significant differences in the physical fitness of girls with different perceptions of physical activity on physical condition. The above results have been shown in Table 4.

The comparison of boys and girls with different perceptions of physical fitness on well-being indicated significant differences in every group. The mean value of physical fitness of adolescents who declared “definitely positive” influence of physical activity on well-being was significantly higher compared to those who declared “irrelevant or definitely negative” influence of physical activity on well-being.

The analysis of physical fitness levels in boys and girls with or without clinically diagnosed postural disorders or lower limb malalignments revealed no significant differences. There were, however, significant differences in physical fitness of boys and the entire population with or without clinically diagnosed foot deformities. In boys who did not report the incidence of foot deformities, the mean value of the physical fitness test was significantly higher compared to boys who reported the incidence of foot deformities. Similar findings were observed when analyzing the results of the entire population. We did not observe significant differences in the physical fitness of girls who reported or did not the incidence of foot deformities. These results have been shown in Table 5.

## 4. Discussion

This study aimed to assess the relationship between SRH and physical fitness in adolescents. The comparison between boys and girls revealed no significant differences in physical fitness levels; there were, however, significant differences in SRH between genders, and the boys reported better SRH than the girls. Similar results were obtained in other studies [25,26]. On the other hand, different results were obtained by Xu et al., who found no significant difference in SRH between genders [27]. A possible explanation for gender differences showing lower SRH among girls might be due to girls tend to be more emotional than boys and may experience concerns related to reproductive issues, give more importance to appearance, body weight, or social relationships. All these factors might influence their self-perception in terms of health state [28].

Also, the incidence of seasonal diseases differed between boys and girls. In this study, the girls reported seasonal diseases more often than the boys. These results are similar to those of Seki et al., who found that the feminine gender was more likely to suffer from influenza [29]. Vom Steeg and Klein suggested that the prevalence of infections is more frequent in males than females [30]. The authors assume that male–female differences in morbidity of seasonal diseases may result from variations in immune responses and hormonal regulation. These physiological disparities may contribute to differences in the control and clearance of a pathogen.

In the present study, there were no significant differences in the incidence of clinically diagnosed postural disorders, lower limb malalignments, foot deformities, sleep duration, and perception of physical activity on physical condition and well-being between boys and girls. The authors assume that no differences in sleep duration may be caused by a fixed lifestyle that is associated with school life. Also, youth may not yet be diagnosed with existing postural disorders, lower limb malalignments, and foot deformities.

Further analysis revealed significant differences in physical fitness levels in youth, with different SRH only in boys. This study found that the physical fitness of the boys who rated their health “very good” was significantly higher than those who rated their health “good” and “poor or bad”. According to Häkkinen et al., boys who declared better health-related quality of life had better physical fitness [31]. Surprisingly, in this study, no differences were found in physical fitness levels with different SRH in girls and the entire population. These findings are somewhat surprising, given the fact that other research showed this relationship in both genders [32]. Further study with more focus on girls is therefore suggested, because of the crucial role of physical fitness in preventing future health issues and premature death. The authors assume that no differences in physical fitness levels with different SRH in girls suggest that SRH may not be an accurate indicator of physical fitness among adolescent girls and the entire population. This lack of significance could be attributed to the substantial number of girls (150 girls vs. 85 boys) and the finding that there is no significant relationship between SRH and physical fitness in Polish girls. This could be also due to not including potential confounders in the analysis. According to the study conducted by Herman et al., physical activity was positively associated with SRH in Canadian adolescent girls only once controlling for confounding variables [33]. Results of other studies also highlight confounders affecting SRH such as pubertal development [34], socioeconomic status [25,34] or nutritional status [35]. Nonetheless, despite the lack of this relationship in adolescent girls and the entire population, promoting physical activity, and encouraging them to engage in regular exercise routines and healthy lifestyles for all of them is crucial, regardless of their SRH.

The authors also investigated whether sleep duration affects physical fitness. The significant differences in physical fitness with different sleep duration in girls and the entire population. Both girls and the entire population who slept 7 to 8 h received higher scores on the physical fitness test compared to those who slept less than 6 h and more than 9 h. These results are consistent with those of Štefan et al., who found that lower levels of physical fitness were associated with poor sleep quality [36]. This has been also confirmed by a systematic review performed by Fonseca et al. [37]. The authors stated that longer periods of sleep, as well as better sleep quality, are associated with higher physical fitness levels. Furthermore, several reports have shown that short sleep deprivation in youth is associated with excessive adipose tissue, poorer emotional regulation and academic performance, and lower well-being [38]. Thereupon, it seems that sleep is crucial in promoting a good health state, and it is appropriate to take care of its correct duration.

In this study, we obtained interesting results, showing that the boys who reported a positive influence of physical activity on physical condition received a higher score of physical fitness. Such a relationship has been seen in the entire population too, but not in girls. We can assume that adolescent girls are not yet aware of the benefits of physical fitness and are not familiar with this area. However, this should be carefully investigated in further research.

Another finding from this study is higher physical fitness in adolescents who declared a positive influence of physical activity on well-being. To the best of our knowledge, no earlier studies have directly investigated the association of physical fitness with a perception of physical activity on well-being in youth. Nevertheless, some studies have shown the positive role of physical fitness in promoting well-being [39] and mental health [40].

In this study, there were no significant differences in physical fitness between boys and girls who reported the incidence or not clinically diagnosed postural disorders or lower limb malalignments. This finding may suggest that these types of conditions do not necessarily impact an adolescent’s overall physical fitness. Despite that, youths who suffer from postural disorders or lower limb malalignments should still receive proper healthcare to maintain a good state of health and prevent potential musculoskeletal complications in the future.

In this study, we also noticed that in boys who did not report the incidence of clinically diagnosed foot deformities, physical fitness was significantly higher. Such a relationship has been seen in the entire population as well. Similar findings were obtained by Abich et al., who also noted that a low level of physical activity was significantly associated with flatfoot [41]. Different results were obtained by Truszczyńska-Baszak et al., who found no significant differences between foot arch disorders with the level of physical fitness [42]. However, the authors emphasize that participants without transversely flat feet were characterized by better physical fitness. Additionally, children whose physical fitness was at a superior level had four times less commonly lowered longitudinal arches.

## 5. Conclusions

There were no differences in physical fitness between Polish boys and girls; however, there were the significant differences in SRH and the incidence of seasonal diseases between these groups. Boys declared better health states and reported less frequent morbidity of seasonal diseases. No associations were found between boys and girls in the incidence of postural disorders, lower limb malalignments, foot deformities, sleep duration, and perception of physical activity on physical condition and well-being. The positive SRH and perception of physical activity on physical condition and well-being, proper sleep duration (7–8 h/night), and lack of foot deformities are associated with a better physical fitness in Polish adolescents. Physical fitness seems to be a good predictor of SRH only in Polish boys, but not girls and the entire population. This lack of significance could be attributed to the substantial number of girls in the entire study population and the finding that there were no significant relationship between SRH and physical fitness in Polish girls. Despite that, it is recommended to encourage Polish adolescents to engage in regular exercises, sleep routines and healthy lifestyles, because healthy behavior in adolescence influences health status in adulthood. To sum up, the present results open new areas for research in this area to fully understand and check the relationship between physical fitness and health. To develop a full picture of the correlation between health and physical fitness in youth, future studies should be based on objectively measured health and a more representative group with a comparable number of girls and boys in the study group.

Further studies will need to be taken and generate stronger evidence concerning the association between health and physical fitness. On the other hand, further research should be conducted on SRH predictors to improve SRH in Polish girls including other factors, such as sedentary behavior, social media screen time or mental health status.

### 5.1. Limitations of the Study

A primary limitation of this study is the absence of an objectively measured health state. The authors are aware that the analysis of medical records, lab results or physical examination would be more beneficial for this study. Despite this limitation, the authors want to point out that SRH is widely used in epidemiological, clinical and social research, and is a predictor of death. Another limitation is the sampling as most of the study participants were girls, and their anthropometric measures were lower compared to boys, thus this could impose constraints on the generalizability of the findings.

### 5.2. Practical Implications

From both practical and scientific point of view, it is important to increase awareness of adolescence in terms of physical fitness and health state. These results may be taken into account by doctors and other clinicians, PE and class teachers, or parents, to primarily identify the adolescents with insufficient physical fitness level and lower SRH and, consequently, allow them to support adolescents by promoting healthy behaviors among them and encourage them to follow healthy lifestyle to improve their health status and avoid future medical complications. The promotion of healthy lifestyle could probably be advertised in social media, as its usage among adolescents is nowadays very high.

## Figures and Tables

**Table 1:** Study group description.

	Gender	Min	Max	Median	Mean (SD)	*p*
Body weight	Girls	42	95	56	57.01 (8.46)	<0.001
	Boys	52	120	68	69.09 (1.51)	
Height	Girls	153	184	166	166.82 (5.64)	<0.001
	Boys	163	190	180	179.25 (5.53)	
BMI	Girls	15.80	32.90	20.20	20.46 (2.62)	0.023
	Boys	15.90	37.00	20.90	21.53 (3.36)	

**Table 2:** Results of Zuchora’s Physical Fitness Index between girls and boys.

Girls		Boys			Normative Data for 16–18 Aged Group
Mean (SD)	Median	Mean (SD)	Median	*p*	6—minimal physical fitness level 12—sufficient physical fitness level 18—good physical fitness level 23—very good physical fitness level 28—high physical fitness level 33—excellent physical fitness level
20.58 (3.59)	20.00	20.19 (3.09)	20.00	0.63	

**Table 3:** The relationship of SRH, the incidence of seasonal diseases, postural disorders, lower limb malalignments, foot deformities, sleep duration, and perception of physical activity on physical condition and well-being in boys and girls.

	Variable	Girls		Boys		*p*
		n	%	n	%	
SRH	Very good	40	29.20%	36	49.32%	0.008
	Good	89	64.69%	36	49.32%	
	Poor or bad	8	5.84%	1	1.37%	
Seasonal diseases	Very often	5	3.65%	2	2.74%	0.038
	Often	24	17.52%	9	12.33%	
	Rarely	84	61.31%	36	49.32%	
	Almost never	24	17.52%	26	35.62%	
Postural disorders	Yes	52	37.96%	25	34.25%	0.595
	No	85	62.04%	48	65.75%	
Lower limb malalignments	Yes	8	5.84%	5	6.85%	0.361
	No	129	94.16%	68	93.15%	
Foot deformities	Yes	11	8.03%	8	10.96%	0.481
	No	126	91.97%	65	89.04%	
Sleep duration	Less than 6 h	53	38.69%	27	36.99%	0.649
	7–8 h	70	51.09%	41	56.16%	
	More than 9 h	14	10.22%	5	6.85%	
Perception of physical activity on physical condition	Definitely positive	72	52.55%	43	58.90%	0.676
	Irrelevant	63	45.99%	29	39.73%	
	Definitely negative	2	1.46%	1	1.37%	
Perception of physical activity on well-being	Definitely positive	92	67.15%	55	75.34%	0.218
	Irrelevant or negative	45	32.85%	18	24.66%	

**Table 4:** The comparison of physical fitness between boys and girls with different SRH, sleep duration, and perception of physical activity on physical condition.

SRH		Very good		Good		Poor and bad		*p*
		Mean (SD)	Median	Mean (SD)	Median	Mean (SD)	Median	
	G	20.88 (3.80)	20.00	20.39 (3.58)	20.00	21.13 (2.75)	22.00	0.628
	B	21.25 (2.55)	21.50	19.17 (3.29)	19.00	19.00 (0.00)	19.00	0.014
	T	21.05 (3.25)	21.00	20.04 (3.53)	20.00	20,89 (2.67)	22.00	0.085
Sleep duration		Less than 6 h		7–8 h		More than 9 h		*p*
		Mean (SD)	Median	Mean (SD)	Median	Mean (SD)	Median	
	G	19.49 (3.37)	20.00	21.50 (3.71)	21.50	20.07 (2.59)	20.50	0.049
	B	19.55 (2.71)	19.00	20.66 (3.12)	21.00	19.80 (4.60)	22.00	0.317
	T	19.51 (3.14)	20.00	21.89 (3.51)	21.00	20.00 (3.09)	21.00	0.02
Perception of physical activity on physical condition		Definitely positive		Irrelevant		Definitely negative		*p*
		Mean (SD)	Median	Mean (SD)	Median	Mean (SD)	Median	
	G	20.86 (3.66)	20.00	20.22 (3.53)	19.50	23.00 (0.00)	23.00	0.366
	B	21.09 (2.72)	21.00	19.03 (3.12)	18.00	15.00 (0.00)	15.00	0.002
	T	20.95 (3.33)	21.00	19.82 (3.44)	19.00	20.34 (4.62)	23.00	0.024

G—girls; B—boys; T—total.

**Table 5:** The comparison of physical fitness in boys and girls with different perceptions of physical activity on well-being, and incidence of postural disorders, lower limb malalignments, and foot deformities.

Perception of physical activity on well-being		Definitely positive		Irrelevant or definitely negative		*p*
		Mean (SD)	Median	Mean (SD)	Median	
	G	20.90 (3.55)	20.50	19.91 (3.62)	19.00	0.101
	B	20.19 (2.82)	21.00	18.28 (3.14)	18.00	0.002
	T	20.87 (3.29)	21.00	19.44 (3.55)	19.00	0.004
Postural disorders		Yes		No		
	G	20,50 (3.52)	20.00	20.62 (3.65)	20.00	0.755
	B	19.84 (2.56)	19.00	20.38 (3.34)	21.00	0.388
	T	20.29 (3.24)	20.00	20.53 (3.53)	21.00	0.542
Lower limb malalignments		Yes		No		
	G	20.30 (2.58)	20.50	20.60 (3.67)	20.00	0.954
	B	20.67 (3.06)	20.00	20.17 (3.11)	20.50	0.788
	T	20.23 (3.00)	21.00	20.46 (3.45)	20.00	0.951
Foot deformities		Yes		No		
	G	19.64 (3.64)	19.00	20.66 (3.59)	20.00	0.240
	B	18.63 (2.00)	18.50	20.38 (3.15)	21.00	0.087
	T	19.21 (3.03)	19.00	20.57 (3.44)	21.00	0.045

G—girls; B—boys; T—total.

## Data Availability

The data presented in this study are available on request from the corresponding author.
